# Understanding carnivore interactions in a cold arid trans‐Himalayan landscape: What drives co‐existence patterns within predator guild along varying resource gradients?

**DOI:** 10.1002/ece3.10040

**Published:** 2023-05-11

**Authors:** Priyanka Justa, Salvador Lyngdoh

**Affiliations:** ^1^ Department of Landscape Level Planning & Management Wildlife Institute of India Dehradun India; ^2^ Academy of Scientific & Innovative Research Ghaziabad India

**Keywords:** camera trapping, competition, diet overlap, niche, occupancy, spatiotemporal partitioning

## Abstract

Predators compete for resources aggressively, forming trophic hierarchies that shape the structure of an ecosystem. Competitive interactions between species are modified in the human‐altered environment and become particularly important where an introduced predator can have negative effects on native predator and prey species. The trans‐Himalayan region of northern India has seen significant development in tourism and associated infrastructure over the last two decades, resulting in many changes to the natural setting of the landscape. While tourism, combined with unmanaged garbage can facilitate red fox (*Vulpes vulpes*), it also allows free‐ranging dogs (*Canis lupus familiaris*), an introduced mesopredator to thrive, possibly more than the native red fox. We look at the little‐known competitive dynamics of these two meso‐carnivores, as well as their intra‐guild interactions with the region's top carnivores, the snow leopard (*Panthera uncia*) and the Himalayan wolf (*Canis lupus chanco*). To study interactions between these four carnivores, we performed multispecies occupancy modeling and analyzed spatiotemporal interactions between these predators using camera trap data. We also collected scat samples to calculate dietary niche overlaps and determine the extent of competition for food resources between these carnivores. The study found that, after controlling for habitat and prey covariates, red fox site use was related positively to snow leopard site use, but negatively to dog and wolf site use. In addition, site use of the dog was associated negatively with top predators, that is, snow leopard and Himalayan wolf, while top predators themselves related negatively in their site use. As anthropogenic impacts increase, we find that these predators coexist in this resource‐scarce landscape through dietary or spatiotemporal segregation, implying competition for limited resources. Our research adds to the scant ecological knowledge of the predators in the region and improves our understanding of community dynamics in human‐altered ecosystems.

## Graphical abstract

The study looks at the competitive dynamics of the red fox, a native mesopredator, and free‐ranging dog, an introduced mesopredator, as well as their interactions with the region's top carnivores, the snow leopard and the Himalayan wolf, in northern India's trans‐Himalayan landscape.

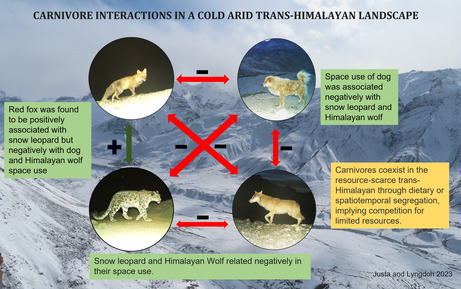

## 1 INTRODUCTION

The populations and geographic ranges of large mammalian carnivores are declining worldwide due to multiple, and sometimes concurring, human threats, including habitat loss and fragmentation, overexploitation, persecution, and prey depletion (Ripple et al., 2014). Slow life histories, wide‐ranging behavior, low population densities, and high energetic constraints make these large carnivores or top predators more vulnerable to anthropogenic influences (Cardillo et al., 2004). Mesopredators, that is, mid‐ranking predators in a food chain (Prugh et al., 2009), however, are likely to benefit from humans owing to their opportunistic behavior which helps them acquire provisioned food resources in the form of garbage, crops, agriculture pests, etc. (Crooks & Soule, 1999; Wangchuk, 2004). Thus, in areas where increasing resource availability is the primary driver of mid‐ranking predators, apex predators are less likely to constrain the distribution, abundance, and behavior of the mesopredators (Prugh et al., 2009). The increase in density, distribution expansion, or the change in behavior of a mid‐rank predator, consequential from a decline in the density or distribution of an apex predator is termed as ‘*mesopredator release*’ (Brashares et al., 2010; Ritchie & Johnson, 2009). Despite its frequent mention in the framework of trophic cascade theory (Berger et al., 2001; Ripple & Beschta, 2007; Terborgh et al., 1999) the phenomenon is primarily an intra‐guild interaction that drives niche partitioning among predators collectively determined by resource acquisition, competitor avoidance, and the response of predators to anthropogenic impacts (Schuette et al., 2013; Smith et al., 2018).

In a carnivore guild, top predators alter the behavior and habitat utilization of mesopredators by killing, instilling fear, or competing with them, ultimately restricting their distribution and abundance (Ripple et al., 2013). Numerically, these interactions manifest in decreased densities, growth, fecundity, and the altered age structure of the mesopredators (MacNally, 1983; Ripple et al., 2013). For example, studies have demonstrated that competition with the wolf (*Canis lupus*) reduces coyote's (*Canis latrans*) abundance, therefore, densities of coyotes appear higher in locations and years where wolf densities are lower (Berger & Gese, 2007; Carbyn, 1982). Similarly, leopard (*Panthera pardus*) populations have been shown to drop in various Indian protected areas following the reintroduction (Mondal et al., 2012) or population recovery (Harihar et al., 2009) of a dominating predator, the tiger (*Panthera tigris*). Studies have also documented intra‐guild predation (IGP), the most extreme way in which top predators suppress mesopredators (Caro, 1987; Donadio & Buskirk, 2006; Palomares & Caro, 1999).

To avoid confrontations and competition, predator guilds adopt various strategies across a range of niche axes including dietary, spatial, and temporal. Selective predation in terms of prey species, size, age, and physical condition has been considered a significant factor in facilitating the coexistence of tigers, leopards, and dholes (*Cuon alpinus*) in many parts of south Indian forests (Andheria et al., 2007; Karanth & Sunquistt, 1995; Ramesh et al., 2009). The coexistence of leopards and lions (*Panthera leo*) in Gir (India), has been demonstrated to be facilitated by site‐specific spatial partitioning and differential preferences for habitat (Chaudhary et al., 2020). Cheetahs (*Acinonyx jubatus*) in the Serengeti have been shown to actively avoid lions (Durant, 2000; Swanson et al., 2016), seeking out ‘competition refugees’ that have low densities of lions and hyenas (*Crocuta Crocuta*) (Durant, 1998). In New Zealand, Stoats (*Mustela erminea*) coexist as the subordinate species of the predatory guild by reducing spatial and temporal exposure to dominant predators namely, feral cats (*Felis catus*) and ferrets (*Mustela furo*) (Garvey et al., 2022). In systems, where sympatric carnivores show high temporal overlaps, they tend to segregate their respective peak activity time as was shown in the case of Oncilla (*Leopardus tigrinus*), Ocelot (*Leopardus pardalis*), and Jaguars (*Panthera onca*) in Brazil (Penido et al., 2017).

The directionality of the competitive interactions among predators is generally determined by body size, with larger carnivores displacing or killing smaller carnivores (Lima & Dill, 1990; Palomares & Caro, 1999). This usual trend can be modified by humans as they have varying impacts on different species (Cardillo et al., 2004; Prugh et al., 2009). Carnivores more tolerant of anthropogenic landscapes may use these areas as refuges from their competitors, that is, the human shield hypothesis (Berger, 2007). It argues that by indirectly benefiting the subordinate competitors, humans facilitate their greater niche partitioning with dominant carnivores (Shannon et al., 2014). Alternatively, the distribution of a species might be likely impacted by its need to reduce encounters with humans, that is, the landscape of fear hypothesis, resulting in fewer opportunities for sympatric carnivores to partition their niche in space and time (Gaynor et al., 2019). Therefore, taking into account anthropogenic impacts is crucial given how much humans are influencing relationships among carnivore groups in the landscapes they have altered (Easter et al., 2020).

The cold desert region of Spiti (India), is one such landscape that has seen rapid infrastructure development, and a boom in tourism in the past two to three decades. (Mishra, 2000; Peaty, 2009). The snow leopard (*Panthera uncia*) and Himalayan wolf (*Canis lupus chanco*) are the top predators, while red foxes (*Vulpes vulpes*) and dogs (*Canis lupus familiaris*) are the mesopredators in the region (Table 1). With a global population of 2710–3386 mature individuals (McCarthy et al., 2017), the snow leopard is threatened by human activities, including habitat loss, retaliatory killings for livestock predation, and poaching for illegal trade (Lovari et al., 2009; Valentová, 2017). The Himalayan wolf also referred to as the Tibetan wolf, or Woolly wolf is a subspecies of the gray wolf (*Canis lupus*) that has evolved a genetic adaptation to cope with the cold and hypoxic environment of high‐altitude ecosystems (Joshi, Lyngdoh, et al., 2020; Joshi, Sharief, et al., 2020; Werhahn et al., 2018). The subspecies have been qualified as an important population and proposed as Evolutionary Significant Unit (ESU) due to its distinct evolution compared to other Holarctic wolf populations (Hennelly et al., 2021). The red fox of the trans‐Himalayan region is a member of the Holarctic clade and belongs to the oldest lineage of the fox population (Statham et al., 2014). Despite thriving well in human habitats, owing to their generalist, opportunist, and highly elusive behavior (Macdonald, 1983), these foxes are rarely sighted and are less studied in the Himalayas (Reshamwala, 2020). Dogs in the region are mostly free‐ranging or feral and are known to prey on local livestock and wildlife (Home et al., 2017, 2018). They kill more livestock in Spiti Valley than both snow leopards and wolves (Suryawanshi et al., 2013). They are also major contributors to livestock losses along with snow leopards and wolves in the trans‐Himalayan landscape of Ladakh (Pahuja & Sharma, 2021).

**TABLE 1:** Ecological characteristics of the study mesopredators (dog and red fox) and top predators (Himalayan wolf and snow leopard) in Lahaul and Spiti, Himachal Pradesh.

Species	Adult weight (kg)	Distribution	Home range size	Diet	Habitat preference	IUCN status	References
Dogs (*Canis familiaris*)	<1 to 70	Worldwide	Variable	Omnivore; varied animal and plant‐based diet	Associated with humans in a wide variety of habitats	Least concern	(Sprain, 1991, Animal Diversity Web)
Red fox (*Vulpes vulpes*)	3–14	Throughout much of the northern hemisphere, Australia, New Zealand	5–12 km^2^ in good areas; 20–50 km^2^ in poorer habitats	Opportunistic omnivores; invertebrates, small mammals, birds, and fruit	Edge habitats, mixed scrub, and woodland	Least concern	(Nowak & Walker, 1999; Sillero‐Zubiri et al., 2004)
Himalayan wolf (Canis lupus chanco)	~35	Himalayan range, Tibetan plateau, extending into Mongolia and China	827–3055 km^2^	Ungulates; small mammals; livestock; carrion	Riversides, marshes (valley habitat); scrub forests	Endangered	(Joshi, Lyngdoh, et al., 2020; Joshi, Sharief, et al., 2020; Khan et al., 2022; Lyngdoh et al., 2019; Shrotriya et al., 2012)
Snow leopard (*Panthera uncia*)	36–52	High mountain ranges of Central Asia	Female: 124 km^2^; Male: 207 km^2^	Mountain ungulates; domestic livestock; small mammals	Steep and rocky cliffs, ridgelines of alpine and subalpine zones	Vulnerable	(Johansson et al., 2015; Mallon et al., 2016; Nyhus et al., 2016)

While there have been some studies on the negative impact of dogs on livestock and wild prey in the Spiti Valley (Home et al., 2017, 2018; Suryawanshi et al., 2013), limited research exists which considers dog's interactions with native carnivores in the entire trans‐Himalayan landscape. Feral dogs with their increasing populations are responsible for a stark decline in the primary prey of the snow leopards in Pakistan (Ahmad et al., 2022). Despite being considered subordinate to wolves, dogs because of their numerical superiority endanger the wild canid through hybridization, disease transmission, and competition (Atickem et al., 2009; Hennelly et al., 2015; Lacerda et al., 2009). Studies have also shown dogs to be a potential competitor and threat to the native red foxes (Ghoshal et al., 2016; Reshamwala et al., 2021).

**FIGURE 1:**
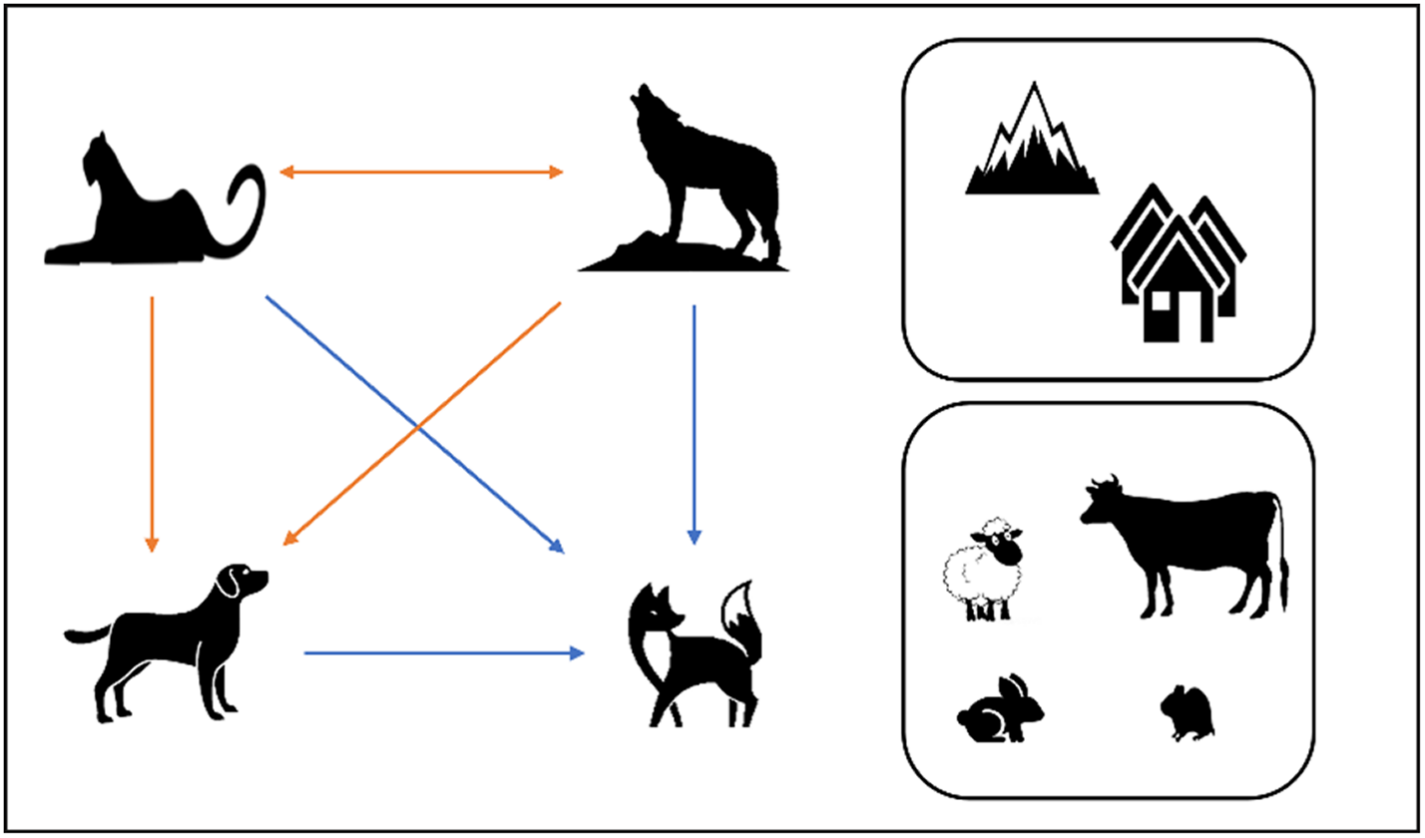
A conceptual framework for predicted interactions of different predator pairs. Arrows in orange and blue arrows denote negative and positive associations respectively. We also study the effect of habitat and prey covariates on multispecies occupancy.

In this study, we examine predatory guild interactions through a multidimensional niche analysis along the axis of space, time, and diet. As species distribution in space is influenced by environmental variables and interactions with other species (Rota et al., 2016), we hypothesize that (1) conditional occupancy of every pairwise combination of the four predators' will be influenced by habitat and prey covariates along with the presence or absence of competing species. In terms of species interaction considering various covariates, we predict, (2) red fox site occupancy to be positively associated with dogs as both species are known to exploit anthropogenic subsidies (Home et al., 2017; Virgos et al., 2002; Webbon et al., 2004). Further, we expect the red fox occupancy to be positively associated with snow leopards and wolves as dominant predators might benefit smaller carnivores by resource provisioning via scavenging, that is, facilitation hypothesis (Ruprecht et al., 2021), (3) dog's site use will be negatively associated with snow leopard and Himalayan wolf given dog's high association with humans (Home et al., 2017, 2018), (4) snow leopard and Himalayan wolf site occupancy will be negatively associated as there is a high potential for the exploitative competition given high dietary overlap (Jumabay‐Uulu et al., 2013; Wang et al., 2014) (Figure 1). Further, we hypothesize (5) the predator pairs will partition their niche across at least one niche axis, that is, spatial, temporal, or dietary to coexist in a human‐altered environment, that is, niche segregation hypothesis (Durant, 1998; Karanth & Sunquistt, 1995), (6) the overlap between predator pairs would be minimum at a site that has a greater human footprint (*human shield hypothesis*) (Berger, 2007) as red foxes and dogs will exploit human resources resulting in lesser overlap with top carnivores. Alternatively, the site with the less human footprint will have high niche overlap between predators leading to more intense competition (*landscape of fear hypothesis*) (Gaynor et al., 2019). To test our hypothesis, we used camera traps to analyze occupancy and spatiotemporal use in three different areas of the valley with varying anthropogenic pressures and species assemblages. We also collected scats to conduct dietary studies on these predators.

## 2 METHODS

### 2.1 Study area

The study area is situated in Himachal Pradesh's Lahaul–Spiti district, with Ladakh to the north, the greater Himalayas to the south and west, and Tibet in the East, having an average elevation between 3900 and 4300 m (Bagchi & Mishra, 2006). The entire Spiti Valley, a trans‐Himalayan high‐elevation landscape, is included in the Cold Desert Biosphere Reserve. (Sharma & Samant, 2019). This Pir Panjal rain shadow region is characterized by extreme climate and temperatures, ranging from brief, dry summers with intense sunlight (maximum temperature reaching 30°C) to long, windy, and freezing winters (lowest temperature reaching – 40°C at night) (Suryawanshi et al., 2014). Alpine scrub or dry alpine steppe is the major vegetation type in this region (Champion & Seth, 1968). Principal mammalian fauna includes snow leopard (*Panthera uncia*), Tibetan wolf (*Canis lupus chanco*), red fox (*Vulpes vulpes*), stone marten (*Martes foina*), Siberian weasel (*Mustela sibirica*), mountain weasel (*Mustela altaica*), blue sheep (*Pseudois nayaur*), ibex (*Capra sibirica*), woolly hare (*Lepus oiostolus*), Himalayan marmot (*Marmota himalayana*), and Royle's pika (*Ochotona roylei*) (Suryawanshi et al., 2014).

The predominantly Buddhist local agro‐pastoral community known as Spitian Bhot or Bodh is concentrated in village clusters primarily along the main Spiti River (Bhasin et al., 1983). There are roughly 60 villages spread out over the length and breadth of the valley, with a total population of 12,445 and the lowest population density (2 people per km^2^) in the country (Government of Himachal Pradesh, 2023). Most agriculture‐related activities are restricted to the short growing season (from May to September). Barley (*Hordeum vulgare*), green pea (*Pisum sativum*), and black pea, a native variation of green pea, are the main crops grown. Sheep (*Ovis aries*), goats (*Capra hircus*), donkeys (*Equus asinus*), cattle (*Bos taurus*), yak (*Bos grunniens*), cow–yak hybrids (dzo, dzomo), and horses (*Equus caballus*) make up the livestock ensemble. Except during peak winter (December to February), when livestock is stall‐fed, livestock is grazed in pastures (Suryawanshi et al., 2014).

### 2.2 Site selection

**FIGURE 2:**
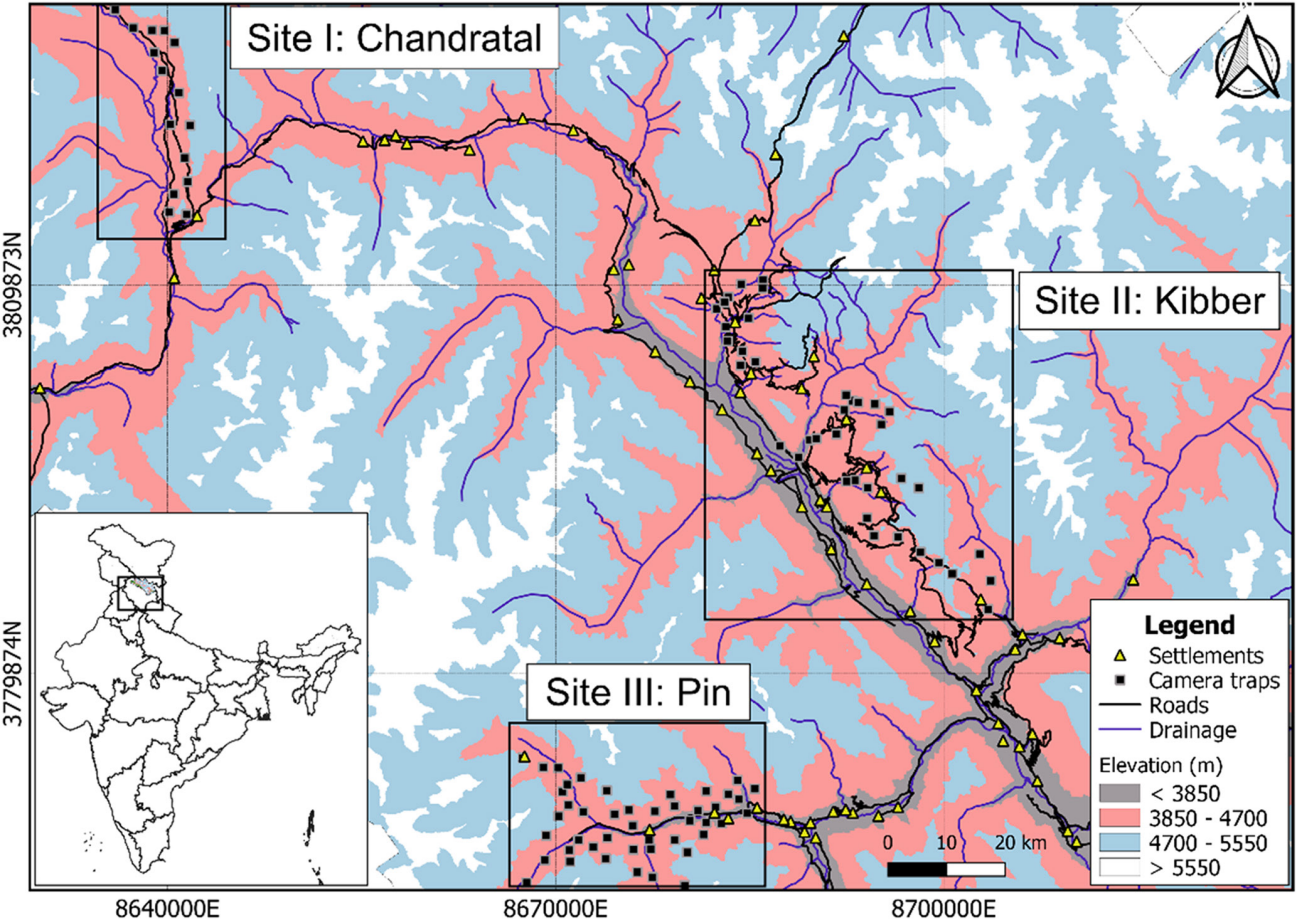
Map depicting three intensive study areas namely Chandratal, Kibber, and Pin in Lahaul & Spiti district, Himachal Pradesh, India.

Based on extant literature, key informant questionnaire surveys (following Ghoshal et al., 2019), and sign surveys, we selected three intensive study locations in the trans‐Himalayan landscape of Spiti Valley (Figure 2). The sites differ in terms of the intensity of anthropogenic imprint, predator and prey distribution and abundance, and the presence of domestic, free‐ranging, and feral dogs.

Chandratal (~60 km^2^) is the source of the Chandra River and a popular hiking and camping destination. The area includes the Chandratal Wildlife Sanctuary and a sacred lake that has been designated as a Ramsar site. The region is also used as a grazing area for livestock during the summer, and it is only open from late May until early October (Dey et al., 2021). Due to its limited accessibility, the area has no permanent settlements and overall is the most pristine of the three, albeit with a greater human footprint during the few months it is accessible.

The research site Kibber (~180 km^2^) lies on the left bank of the Spiti River and includes principal settlements of the valley as well as the administrative headquarters Kaza. Over the years, tourism infrastructure has grown in many locations within this area and has become a significant source of revenue for the local communities. While tourism has benefited the locals, an increase in the associated garbage has resulted in large stable populations of dogs in Kaza and the surrounding villages (Home et al., 2017). The terrain is rolling, interrupted by a few cliffs. The blue sheep is the most common wild ungulate here, with a small population of ibex (Bagchi & Mishra, 2006). This study site exhibits the largest human footprint of the three.

The third site, Pin (~140 km^2^) comprises the catchment of the Pin and Parahio rivers and most of it forms a part of the Pin Valley National Park (PVNP). There are four *dogharies* (summer settlements) in the core zone, and herders use the area as grazing grounds in the summer. The area comprises the habitation and pastures of Mud and Sagnam villages. Locals depend on the national park area for agriculture, fuelwood, and grazing of their livestock (Sharma, 2008). The terrain is rugged and most of the area inclines between 30° and 60°. PVNP has a single wild ungulate, the ibex *Capra sibirica* (Bagchi & Mishra, 2006). On the disturbance gradient, this site sits between Chandratal and Kibber.

### 2.3 Field surveys

To study the spatiotemporal interactions of the top predators (snow leopard, Himalayan wolf) and mesopredators (red fox, dogs) we deployed a total of 97 camera traps in a 1 × 1 sq. km grid in the three selected study sites: 15 in Chandratal (August 25, 2021, to October 2, 2021), 39 in Kibber (October 12, 2021, to November 30, 2021) and 43 in Pin Valley (September 7, 2021, to October 15, 2021). In grids that were accessible, camera traps were randomly placed on one among the many trails or in a location away from a trail with carnivore signs (Marinho et al., 2018). Focusing on signs associated with carnivores and camera placement on trails is particularly important for relatively uncommon or low‐density species that are hard to detect (Kolowski & Forrester, 2017; Tanwar et al., 2021). A single camera with motion sensors was deployed at each location, and a time lag of 2 s was set between animal detections. Cameras were fastened to rock piles at the height of 30–45 cm above the ground for an average of 35 days in Chandratal and Kibber respectively and 32 days in Pin. After the completion of one camera‐trapping session across three sites, the photographs were examined for images of animals using a field guide (Menon & Daniel, 2003). The amount of trapping effort required (unit: camera days) was calculated for each camera from when the camera was mounted until the camera was retrieved. The total trapping effort in a sampling period was 3278 days which included camera days of all cameras across Chandratal (529 days), Kibber (1355 days), and Pin (1394 days).

Scat samples of the study carnivores were collected from the field during the sign survey (trail transects) and opportunistically while walking on trails for deploying the camera traps in Aug – Nov 2021. Scats of the study carnivores were distinguished in the field based on shape, size, odor, quantity, and signs typical to that of the relative species (Jackson & Hunter, 1996; Menon, 2003; Vanak & Mukherjee, 2008). Studies solely based on the visual identification of samples may be strongly biased because of field misclassification of species (Jumabay‐Uulu et al., 2013; Shrestha et al., 2018; Weiskopf et al., 2016). In our study, confusion about the predator's identity (snow leopard, wolf, and dog) might have occurred. However, wolves use relatively flat and rolling terrain (Khan et al., 2022; Lyngdoh et al., 2019) while snow leopards use ridges and high cliffs (Watts et al., 2019) and all the dog scats were collected from human habitation. Therefore, the confusion between scats of snow leopards, wolves, and dogs can be considered to be minimal in this study. Further red fox scats were easily distinguished due to their small size and quantity (Vanak & Mukherjee, 2008). All of the scat samples were collected in a zip lock bag and were labeled with sample ID, date, GPS location, and habitat characteristics. The scats which had moisture were sun‐dried till they were completely dry for storage.

### 2.4 Data analysis

#### 2.4.1 Patterns in space use: Multispecies occupancy modeling

##### Covariates

We modeled variation in occupancy using five covariates considering previous studies (Contardo et al., 2021; Ghoshal et al., 2019; Murdoch et al., 2016; Rehman et al., 2021) (Table S1). We hypothesized that the distribution of predators would be influenced by habitat including natural and anthropogenic factors and available prey resources. We selected elevation (DEM) and NDVI as habitat covariates. The effect of anthropogenic impacts was tested with Distance to roads and settlements. The encounter rate of prey species at each camera station was taken as a prey covariate which was calculated by evaluating trap success (number of detections/total camera trap days × 100) per species (Merson et al., 2020). The encounter rates of small prey, namely pika and the woolly hare, were considered for the conditional occupancy modeling for red fox, whereas the encounter rates of large prey, namely Himalayan ibex, blue sheep, and livestock, were considered for the dog, snow leopard, and Himalayan wolf. The covariates were averaged out for each camera trap grid (survey site) using the spatial analyst tool in QGIS 3.16.14. We used trap nights as a detection covariate. All continuous variables were standardized to *z* scores before analysis. We diagnosed univariate correlations using a Pearson correlation matrix and dropped correlated covariates (*r* > .4) (Hooten et al., 2015).

#### 2.4.2 Modeling framework

We used the multispecies occupancy model (Rota et al., 2016), to access the spatial interaction of dogs, red foxes, snow leopards, and wolves while accounting for imperfect detection. This model is a generalization of the single‐species occupancy model (MacKenzie et al., 2017) that accommodates two or more interacting species. For each of the species, *s* = 1, …, *S* at sites *I* = 1, …, *I*, a latent occupancy state (*Zi*) is included in the modeling framework represented by a sequence of 0/1 values (indicating species absence/presence). The latent state is modeled using a multivariate Bernoulli distribution, *Zi* ~ MVB(*ψi*), where *ψi* is a function of (2^S^−1) natural parameters (*f*) which can all be modeled as linear functions of covariates. It describes the probability of a site being occupied by only one species (first order); only two species (second order); and up to order S, the number of interacting species. If all second‐order and higher natural parameters are set to 0, then species occurrence is assumed to be independent. Whereas, dependence between species can be modeled by estimating the parameters associated with second‐order and higher natural parameters, where slope coefficients associated with first‐order natural parameters can be interpreted as log odds ratios of occupancy probabilities (i.e., the log of the probability a species occupies a site divided by the probability it does not) resulting from a one‐unit change in a relevant covariate (Rota et al., 2016).

For the multispecies occupancy analysis, we formatted the camera trap data by generating detection histories into matrices of sites *i* by sampling occasions *j*, using 1 week as the resolution for model convergence and precision of estimates using the R version 4.2.2 package camtrapR (Niedballa et al., 2016). In our analysis, we only considered pairwise interactions between four predators resulting in 10 natural parameters being modeled. Out of 10 natural parameters, four (*f*_*i*_ = *f*_1_, *f*_2_, *f*_3_, *f*_4_) represented the log odds of a single species occupying a site, and the other six (*f*_*i*_ = *f*_12_, *f*_13_, *f*_14_, *f*_23_, *f*_24_, *f*_34_) were the log odds of the probabilities that two species occur together, with numbers 1–4 denoting species identity. Given that the multi‐species co‐occurrence model can compute both single‐species occurrence and co‐occurrence parameters *f* as a function of covariates, we used a two‐step approach (Salvatori et al., 2021). In the first step, we selected the most supported covariates for single species. In the second step, the top covariates from single‐species occupancy models were included as predictors of occurrence and detectability of single species in the multi‐species model. Due to the complexity of these models, we limited models that tested the effects of variables to no more than two biologically relevant covariates. In total 15 models were run per species pair and we selected the best models by comparing the Akaike information criterion corrected for a small sample size (AICc), Akaike weight (AICc weight), and significance of beta estimates of the models within 2 AICc (Akaike, 1974; Burnham & Anderson, 2002) (Table S2). We implemented the models using the package “unmarked” and “AICcmodavg” in R (Fiske & Chandler, 2011; Mazerolle, 2023). We acknowledge that the closer positioning of the cameras caused the spatial units to become non‐independent due to the wolves' and snow leopards' extensive ranging patterns. As a result, the study's occupancy estimates serve as a proxy for “space use” rather than the matrices of “abundance” or “distribution” and co‐occurrence dynamics may change depending on the spatial scale sampled (Burton et al., 2015).

### 2.5 Spatial overlap

Spatial overlap between species was assessed through Pianka's niche overlap index (Pianka, 1973). For calculating the index, we used the relative abundance index (RAI) (Carbone et al., 2001; O'Brien et al., 2003) of the four predator species. Photographic rate is the relative index of the animal's spatial use and a crude abundance estimate (Carbone et al., 2001) hence, we treated each camera trap station as spatially independent to compute RAI and the spatial overlap index. Photographic rate as an index of animal abundance is useful because rigorous techniques like mark‐recapture cannot be applied to species like the Himalayan wolf and red fox as they cannot be distinguished based on their markings. Further, the index can be used as an alternative for cryptic, widespread, and low‐density carnivores such as Himalayan wolves where it can be challenging to obtain accurate abundance estimates (Carbone et al., 2002). The spatial overlap index measures the relative amount of habitat overlap between each pair of species and ranges from a minimum of 0.0 (no shared habitats) to a maximum of 1.0 (identical habitat use) (Pianka, 1973).

### 2.6 Temporal overlap

The date and time printed on the photographs were used to determine the activity periods of each species. We assumed that the number of camera trap records taken at various times was correlated with the daily activity patterns of mammals (Linkie & Ridout, 2011). To maintain statistical independence and reduce bias caused by repeated detections of the same individual, one record of each species per half hour per camera trap site was considered an independent detection, and subsequent records were removed (O'Brien et al., 2003). The analysis was performed for the predators with at least 10 records. We used kernel density estimation to estimate the overlap coefficient, a quantitative measure ranging from 0 (no overlap) to 1 (identical activity patterns), in the R platform using the overlap package (Ridout & Linkie, 2009). Overlap coefficient (Δ) was defined as the area under the curve that is formed by taking at least two density functions at each time point ranging from 0 (no overlap) to 1 (complete overlap), where 0 implies that the species have no common active period and 1 implies that the activity densities of two species are identical (Schmid & Schmidt, 2006). The precision of this estimator was obtained through a 95% confidence interval (CI), as percentile intervals from 999 bootstrap samples (Linkie & Ridout, 2011; Meredith & Ridout, 2016).

### 2.7 Diet

The collected scat samples were processed in the Animal Biology lab, Dehradun for diet analysis of the carnivores from December 2021 to January 2022. Scats were cleaned with a soft brush, weighed, and measured (length and diameter). The sample was then homogenized and undigested items (hair, bone fragments, hooves, feathers, claws, chitin remains of insects, seeds, and other plant material) and human‐derived materials (cloth, paper, and plastic) were collected in labeled zip lock bags for further identification. After separating various items, samples of 20 hairs from each scat were randomly drawn for prey identification (Jethva & Jhala, 2003; Mukherjee et al., 1994). Microscopic slides of randomly picked hair from a sample were washed in hydrogen peroxide and xylene and examined under a light microscope to identify prey consumed by medullary hair patterns (Anwar et al., 2012; Bahuguna et al., 2010; Karanth & Sunquistt, 1995; Oli, 1993). Due to limited scat samples, we also included previously published and unpublished scat data for snow leopards (Bagchi & Mishra, 2006), wolves (Lyngdoh & Habib, 2019), and red foxes (Reshamwala et al., 2018) from the same study sites as ours for the analysis. We dropped dog diet analysis in this study due to limited samples.

We calculated the relative frequency of occurrence (RF) as no. of occurrences of each food type (species) when present/total no. occurrences of all food items × 100 (Shrestha et al., 2018). To calculate food niche overlap between the carnivores, we used Pianka's index, with the formula:

$$O_{\mathrm{jk}}=\frac{\sum P_{\mathrm{ij}}\times \sum P_{\mathrm{ik}}}{\sum {P_{\mathrm{ij}}}^{2}\times \sum {P_{\mathrm{ik}}}^{2}}$$

where *O*_*jk*_ is Pianka's index of niche overlap between species *j* and *k*, while *P*_*ij*_ and *P*_*ik*_ are the proportions of use of resource category *i* by species *j* and *k*, respectively. We took prey species to be a resource category. Pianka's index varies between 0 (total separation) and 1 (total overlap) (Krebs, 1989; Pianka, 1973).

## 3 RESULTS

### 3.1 Spatial interactions

#### 3.1.1 Patterns in space use: Multispecies occupancy modeling

A total of 3278 camera days resulted in 64, 309, 72, and 14 independent captures of dog, red fox, snow leopard, and wolf, respectively. Estimated naïve occupancy (i.e., the proportion of sites in which the predators were detected) was 0.3 for dogs, 0.7 for red fox, 0.4 for the snow leopard, and 0.1 for the wolf. According to null models, the detection probabilities of dog, red fox, snow leopard, and Himalayan wolf were 0.1 ± SE 0.05, 0.5 ± SE 0.03, 0.2 ± SE 0.04, and 0.2 ± SE 0.09 respectively. The null model performed better than the models that considered trap nights as detection covariate. The marginal occupancy probability of dogs was best explained by prey covariate where the occupancy probability of dog increased with an increase in the prey encounter rate. Whereas, the habitat covariates best predicted the marginal occupancy probabilities of red fox, snow leopard, and Himalayan wolf. The occupancy probabilities of red fox and snow leopard declined with an increase in distance from the road and higher elevation increased the probability of a wolf's presence (Figure S1).

**FIGURE 3:**
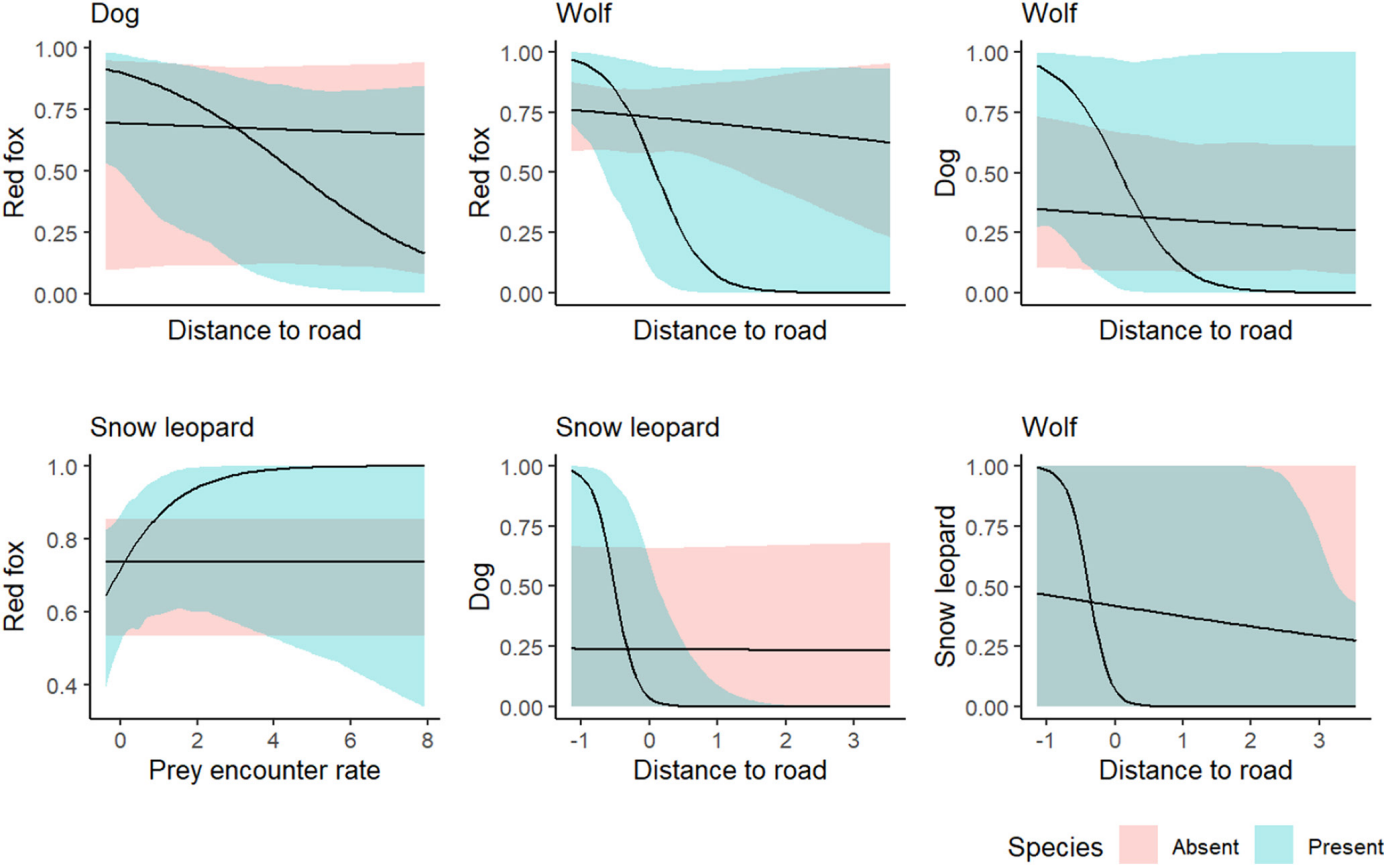
The occupancy probability of red fox, dog, and snow leopard (on the *Y* axis) is conditional on the presence and absence of each of the other species (mentioned on top of each graph) given top model covariates. Lines represent posterior means, and ribbons envelop 95% credible intervals. All variables not included in a plot are assumed fixed at their observed mean. Additionally, conditional plots are marginalized over the two species that do not occur in a plot.

Without taking into account covariates, pairwise interactions were found to be statistically non‐significant (Table S2). However, when habitat or prey covariates were taken into account, we observed some statistically significant pairwise interactions among the four carnivore species (Table 2). The conditional occupancy probability of red fox with snow leopard was best explained by the prey covariate, where the occupancy probability of red fox increased with an increase in the encounter rate of prey when snow leopard was present. Conditional occupancy of other predator pairs was best explained by habitat covariates. The red fox was less likely to occupy a location as the distance to the road increased given the presence of dogs and wolves. Similarly, the probability of a dog occupying a site conditional on snow leopard or wolf presence decreased with increasing distance to the road. The likelihood of snow leopards occupying a site given wolf presence also declined with an increase in distance to the road. Further, given the presence of wolves and snow leopards, the dog's occupancy probability decreased with an increase in NDVI. Snow leopard occupancy probability also showed a declining trend with an increase in NDVI given the wolf's presence. The predictions of each species' site‐use probability conditional on the presence or absence of the interacting species, given the best covariate derived from the multi‐species co‐occurrence model, are shown in Figure 3.

**TABLE 2:** Covariate coefficient estimates from the multi‐species co‐occurrence model for dog, red fox, snow leopard, and wolf in Lahaul and Spiti, H.P., India.

Species pair	Estimate	SE	z	*p* (>|*z*|)
[Red fox: Dog] Distance to road	−1.2	0.8	−1.5	.14
[Red fox: Snow leopard] Prey	0.9	0.6	1.4	.15
[Red fox: Wolf] Distance to road + Prey	−3.7	2.0	−1.9	.06*
	−9.4	8.2	−1.1	.25
[Dog: Snow leopard] Distance to road + NDVI	−10.9	3.8	−2.8	.00**
	−1.5	0.7	−2.3	.02**
[Dog: Wolf] Distance to road + NDVI	−10.2	4.5	−2.3	.02**
	−2.2	1.0	−2.1	.03**
[Snow leopard: Wolf] Distance to road + NDVI	−11.4	3.6	−3.0	.00**
	−1.5	0.6	−2.4	.02**

*Note*: All second‐order models are conditioned on all other species being absent. Asterisks indicate significance levels, one asterisk stands for 10%, two asterisks indicate 5%.

#### 3.1.2 Spatial overlap

Spatial overlap between apex predators and mesopredators ranged from 0 (between snow leopard and free‐ranging dog in Pin) to 0.9 (between the Himalayan wolf and red fox in Chandratal). Overlap between red fox and dogs, mesopredators in all three areas ranged from 0.2 in Pin to 0.6 in Chandratal, while it was 0.5 in Kibber (Table 3).

**TABLE 3:** Spatial overlap between species pairs across three study sites, Pianka Index: Observed (95% CI) − indicates no/less than 10 captures of one of the species pair.

Species pair	Chandratal	Kibber	Pin
Snow Leopard – Red Fox	–	0.4 (0.2–0.7)	0.4 (0.2–0.6)
Snow Leopard‐ Free‐ranging dog	–	0.3 (0.1–0.6)	0 (0)
Free‐ranging Dog – Red Fox	0.6 (0.2–0.9)	0.5 (0.1–0.7)	0.2 (0.1–0.4)
Himalayan wolf – Free‐ranging dog	0.7 (0.0–0.9)	–	–
Himalayan Wolf ‐ Red Fox	0.9 (0.7–1.0)	–	–

### 3.2 Temporal interactions

**FIGURE 4:**
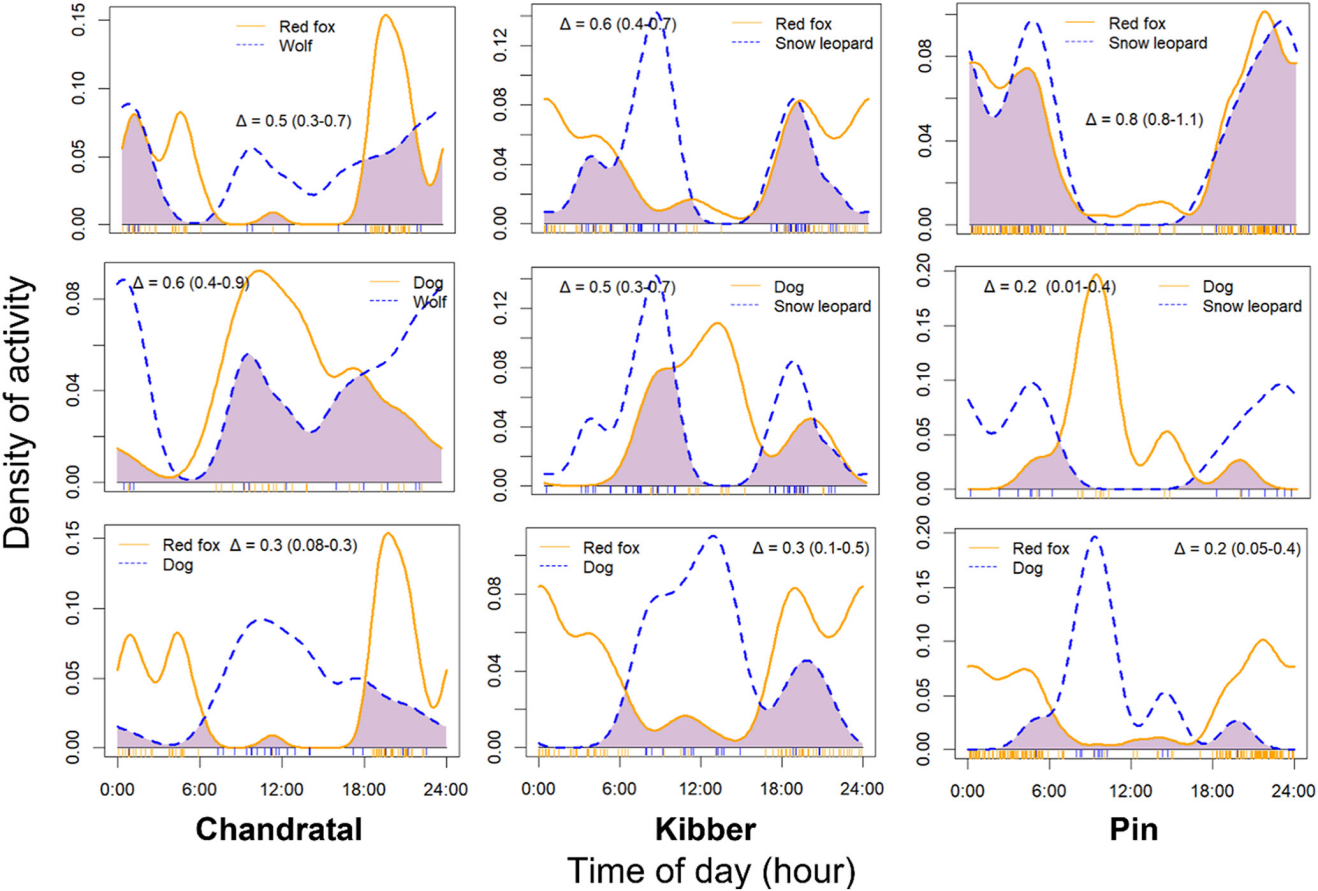
Overlap coefficients of activity patterns and 95% confidence intervals: in brackets () between predator pairs across three study sites.

The mesopredators, dogs, and red foxes, displayed different temporal patterns of activity with distinct activity peaks (Δ = 0.3 in Chandratal and Kibber; Δ = 0.2 in Pin). Regarding interactions between mesopredators and top predators, red fox activity peaked at a different time than wolf's in Chandratal (Δ = 0.5). Red fox and snow leopard activity overlap was found to be moderate (=0.6) and high (=0.8) in the respective regions of Kibber and Pin. Furthermore, there was moderate (=0.5) to low (=0.2) temporal activity overlap between the dog and the snow leopard in Kibber and Pin, respectively (Figure 4). Due to limited captures of wolves, temporal overlaps between top predators could not be analyzed.

### 3.3 Diet

A total of 80 carnivore scats were analyzed for this study which includes 32 from Chandratal, 10 from Kibber, and 38 from Pin. Additionally, we combined the previous dataset for the present analysis with 44 and 51 scat samples of snow leopards from Kibber and Pin, 25 and 50 scat samples of the wolf from Chandratal and Kibber, and 127 red fox scat samples from Kibber.

For determining the relative frequency of various food items in the diets of predators and mesopredators across three study sites we analyzed the remains of 4 domestic and 7 wild species. The diet of the snow leopard, which is one of the top predators in all three study sites, consisted mostly of domestic animals across Chandratal and Kibber (~45%) and wild animals (~44%) in Pin. A major portion of the wolf's diet was constituted by domestic animals in both Chandratal (71%) and Kibber (66%). Further, a major portion of the red fox's diet (>25%), consisted mostly of lagomorphs and rodents across all three study sites (Table 4).

**TABLE 4:** Relative frequency of different kinds of food items in the diet of carnivores in the three study areas: Chandratal, Kibber, and Pin.

	Chandratal			Kibber			Pin^a^	
	Snow leopard	Red fox	Wolf	Snow leopard	Red fox	Wolf	Snow leopard	Red fox
No. of samples (*N*)	12	16	29	47	134	50	59	30
Food items	Relative frequency (%)							
Wild animals	**37.9**	**58.7**	**14.4**	**51**	**52.3**	**26.8**	**44.3**	**39.4**
Blue sheep	0.0	0.0	0.0	**19.6**	4.6	7.1	0.0	0.0
Ibex	3.4	0.0	2.9	7.8	0.0	3.6	**41.4**	1.0
Marmot	6.9	8.7	0.0	2.0	0.0	1.8	0.0	0.0
Stone marten	0.0	2.2	0.0	0.0	0.0	0.0	0.0	1.0
Mountain Weasel	0.0	6.5	0.0	0.0	0.0	0.0	0.0	0.0
Lagomorph (Woolly hare, Pika)	6.9	6.5	0.0	5.9	**24.2**	0.0	2.9	10.1
Rodent (Vole, House Rat)	**13.8**	**19.6**	8.6	2.0	15.0	**12.5**	0.0	**14.1**
Bird	6.9	**13.0**	2.9	**13.7**	2.6	1.8	0.0	7.1
Insect	0.0	2.2	0.0	0.0	5.9	0.0	0.0	6.1
Domestic animals	**44.8**	**23.9**	**71.4**	**45.1**	**22.9**	**66.1**	**42.9**	**11.1**
Goat	17.2	4.3	20.0	9.8	9.2	12.5	8.6	**4.0**
Sheep	6.9	4.3	17.1	7.8	0.0	8.9	7.1	3.0
Yak/Cattle (Cow/Dzo)	**20.7**	13.0	34.3	11.8	**13.1**	**41.1**	11.4	3.0
Horse/Donkey	0.0	2.2	0.0	**15.7**	0.7	3.6	**15.7**	1.0
Others	**17.2**	**17.4**	**14.3**	**3.9**	**24.9**	**7.2**	**12.8**	**49.5**
Plant material (leaf/grass/stem/seed)	**13.8**	**13.0**	11.4	3.9	**19.6**	**3.6**	**11.4**	**44.4**
Plastic/paper	0.0	2.2	0.0	0.0	4.6	0.0	1.4	0.0
Unidentified	3.4	2.2	2.9	0.0	0.7	3.6	0.0	5.1

*Note*: The most frequently consumed prey by the predators is marked in bold.

^a^ No wolf samples were found from Pin.

**FIGURE 5:**
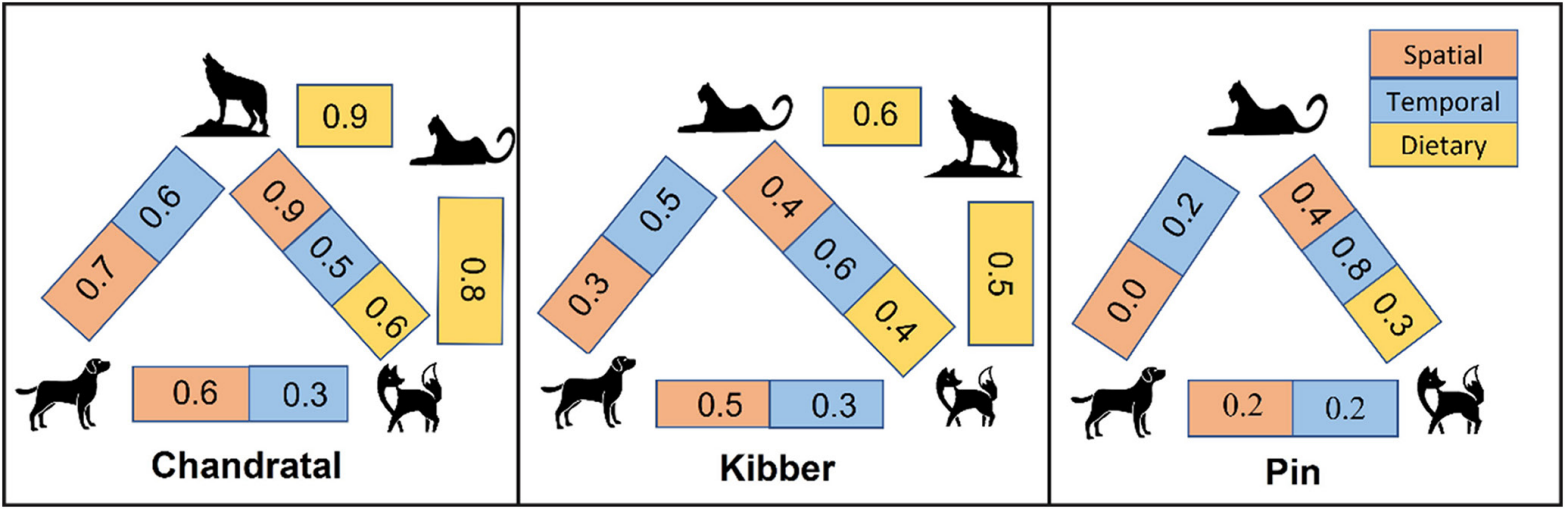
Spatial, temporal, and dietary niche overlap coefficients between free‐ranging dogs, red foxes, snow leopards, and Himalayan wolves across three study sites.

Dietary overlap was calculated for three species pairs in Chandratal and Kibber. In Chandratal snow leopard and wolf (0.9) had maximum diet overlap, followed by snow leopard and red fox (0.8), and the minimum overlap was for wolf and red fox (0.6). In Kibber maximum diet overlap was between snow leopard and wolf (0.6), followed by red fox and wolf (0.5) and the minimum overlap was for snow leopard and red fox (0.4). In Pin, diet overlap could only be calculated for snow leopards and red foxes (0.3) (Figure 5).

## 4 DISCUSSION

Our study provides insights into understudied carnivore intra‐guild interactions in a resource‐scarce trans‐Himalayan landscape. The results of multispecies occupancy modeling showed that co‐occurrence patterns between various predator pairs were primarily negative. As hypothesized, model inference for pairwise occupancy estimates improved after adding either the habitat or the prey covariate (Table S3). This is similar to a study by Rota et al. (2016) where the occupancy probabilities of bobcats, coyotes, gray foxes, and red foxes were related both to environmental variables and the presence or absence of other interacting species. Another study from Bhutan demonstrates that leopard, dhole, and tiger co‐occupancy probability was related to habitat covariate of human settlement density and prey abundance (Penjor et al., 2022). On the contrary, while habitat and preferred prey influenced the occupancy of Puma, Bobcat, and Coyote in New Mexico, USA, the presence of other intraguild predators did not influence the occupancy of these species (Bender et al., 2017).

Our hypothesis that red fox and snow leopard occupancy probabilities would be positively correlated was supported by the findings of this study, which showed that the log odds of red fox being present at a site increased with an increase in prey encounter rate conditional on snow leopard presence. However, contrary to our hypothesis log odds of red fox occupancy given dog presence decreased with increasing distance to the road indicating their negative co‐occurrence patterns. Despite being statistically insignificant, the inclusion of prey species as a top model covariate in the case of red fox occupancy may highlight the importance of wild prey species for a predator that is typically thought of as a generalist species (Macdonald & Reynolds, 2008). The importance of wild prey to the red fox is also apparent as small mammals (lagomorphs and rodents) constituted a major portion of the red fox's diet in our study area. The positive association between red fox and snow leopard might also be attributed to the carrion scavenging by the red fox, increasingly being recognized as a successful mesopredator strategy (Prugh et al., 2019; Sivy et al., 2017). A decrease in red fox occupancy with increasing distance to the road given dog presence may signify interspecies competition between the two. Dogs are known to harass red foxes (Home et al., 2018; Vanak & Gompper, 2010) because of which foxes might be avoiding dogs. However, a previous study in the area has found red fox occurrence to be positively associated with the density of dogs in winter (Ghoshal et al., 2016), indicating that the interaction between red fox and dog might vary with season an aspect that warrants further study. Further, contrary to our expectation, the occupancy probability of the red fox declined given an increase in distance to the road conditional on wolf presence. This might have to do with the wolf not being as tolerant of humans as the red fox, which is known to even den in sites closer to roads and human settlements (Reshamwala et al., 2021).

In consistency with our predictions, the dog's occupancy probability given snow leopard and wolf presence, and occupancy probability of snow leopard given wolf presence were found to be negatively associated with an increase in distance to road and NDVI as top model covariates. Dogs are largely associated with humans in a wide variety of habitats (Contardo et al., 2021; Home et al., 2017, 2018). A previous study conducted in the upper Spiti landscape has shown the density of free‐ranging dogs to be positively related to village size and garbage availability (Ghoshal et al., 2016). On the contrary, snow leopard and wolf are wild predators with the former preferring high ruggedness areas with cliffs and steep slopes (Watts et al., 2019) while the latter is associated with valleys and flat areas of high mountains (Khan et al., 2022; Lyngdoh et al., 2019). Previous studies have shown snow leopard occupancy to be positively related to terrain ruggedness index and altitude (Ghoshal et al., 2019) and wolf occupancy to increase with increasing distance to settlement (Salvatori et al., 2021). Therefore, differences in site use of these predators might have resulted because of their differential habitat preferences apart from all the predators co‐occurring near roads. Roads in the area do not appear to be a disturbance factor since they are mostly dirt trails and non‐tar roads with few vehicles due to inaccessibility, and these carnivores tend to use low‐traffic roads and trails, as demonstrated by other studies (Paschoal et al., 2018; Reshamwala et al., 2021; Whittington et al., 2005). Although our models are useful for illustrating the co‐occurrence patterns of the study predators, given the wide confidence intervals, we are unable to make any firm conclusions about the ecological interactions. This remains a caveat and calls for a long‐term study as low photo‐capture rates of low‐density cryptic carnivores make it challenging and, in some cases, impractical to collect enough samples (Blanchet et al., 2020).

Species within a guild vary in terms of using space, time, and resources (Garvey et al., 2022; Hayward & Slotow, 2009; Karanth et al., 2017; Penido et al., 2017). Numerous studies point to spatio‐temporal partitioning as a crucial tactic for successful carnivore coexistence (Sierra et al., 2023; Vinitpornsawan & Fuller, 2020; Zhao et al., 2020). It allows smaller species to avoid confrontations and agonistic interactions with dominant predators (Tsunoda et al., 2018). Low spatiotemporal overlap between red foxes and dogs, suggests that dogs may be limiting red fox resource use, causing the latter to avoid the former, as seen in previous studies (Silva‐Rodríguez et al., 2010; Vanak & Gompper, 2010). Red fox shows limited spatial overlap with snow leopards in both Kibber and Pin. Whereas, it has high temporal overlap with snow leopards in Pin and moderate overlap in Kibber where red foxes are found nocturnal and snow leopards crepuscular. High temporal overlap (Δ = 0.7) between the two species has also been reported from the Qionglai mountains of Sichuan Province, (Shi et al., 2021) and Qilianshan National Nature Reserve, China (Alexander et al., 2016). Similarly, in Chandratal, wolf and red fox have a high spatial overlap but a lower temporal overlap. On the contrary, a study in Italy detected substantial temporal overlap between the wolf and red fox (Rossa et al., 2021). Substantial interspecific overlap between top predator and mesopredator either temporally or spatially might indicate the facilitation of the latter by the former (Bassi et al., 2012; Codron et al., 2018; Rossa et al., 2021). Dogs have little spatio‐temporal overlap with snow leopards in both Kibber and Pin. In general, dogs were diurnal, a pattern that has been reported elsewhere (de Cassia Bianchi et al., 2020; Farris et al., 2015). Human activities and provisioning are known to strongly influence the diurnal activity of dogs (de Andrade Silva et al., 2018), on the other hand, humans and dogs may constrain the temporal niche of native carnivores as seen in other studies (Wang et al., 2015).

Spatio‐temporal segregation is more likely when competing species have similar diets, with smaller species avoiding the larger one (e.g., Donadio & Buskirk, 2006; Palomares et al., 1995). Both dogs and red foxes are known to subsist on human‐derived foods indicating direct competition (Reshamwala et al., 2018; Vanak & Gompper, 2009). Red foxes in our study primarily survived on the small mammal diet. The consumption of ungulates by red foxes may be explained by them scavenging on the carcasses left behind by larger predators, which serve as a valuable source of food for smaller predators (Bassi et al., 2012; Codron et al., 2018). Further, the consumption of anthropogenic food subsidies by red foxes is known to decline in areas where dogs are present (Linnell & Strand, 2000; Reshamwala et al., 2018; Vanak & Gompper, 2010). This may be the case in the current study and calls for further research as we were unable to account for the impact of dogs on the dietary ecology of red foxes in the present study due to a lack of dog scat samples or earlier research from the landscape on this aspect. Considering red fox overlap with the top predator, it shows sizable dietary overlap with snow leopard and moderate overlap with the wolf in Chandratal. Here small mammals contribute majorly to the snow leopard diet, most likely due to the lower density of its major prey species resulting in a high diet overlap with red fox (Chundawat & Rawat, 1994). In contrast, higher ungulate densities in Kibber and Pin could explain the lower dietary overlap between red foxes and snow leopards in these sites (Bagchi & Mishra, 2006).

Dietary overlap between snow leopards and wolves was found to be high in both Chandratal (0.9) and Kibber (0.6), the sites where the two species co‐occur. Livestock comprised almost 40%–50% of snow leopard's diet across all three study sites, with even a larger contribution to wolves' diet. Our findings are similar to those from the Karakoram Mountains of Pakistan, where livestock made up to 60% and 75% of the diets of snow leopards and wolves respectively, resulting in a high dietary overlap of 0.7 (Bocci et al., 2017). A study from Kyrgyzstan, on the other hand, found no livestock remains in snow leopard and wolf scats and a very high diet overlap (0.9) due to their sharing of limited wild prey species (Jumabay‐Uulu et al., 2013). Livestock depredation in this landscape by snow leopard has been explained by its relative abundance and that of its prey, while habitat structure has been identified as a major factor determining livestock kills in the case of the Himalayan wolf (Suryawanshi et al., 2013).

Although spatiotemporal and dietary niche overlaps between predator pairs varied across three study sites, we cannot conclusively say if our results support the human shield hypothesis (Berger, 2007) or the landscape of fear hypothesis (Gaynor et al., 2019) due to data gap in terms of certain predator pairs overlaps across all niche axis. We do find greater overlap coefficients in Chandratal, a site with comparatively lesser human disturbance compared to Kibber and Pin where overlap coefficients are lower (Figure 5). We can, therefore broadly assume that the results gravitate more toward the human shield hypothesis (Moll et al., 2018; Shannon et al., 2014), where mesopredators, that is, dogs and to some extent, foxes might be benefiting from living close to humans exploiting anthropogenic resources. Although, this aspect still requires longer‐term research.

Land use changes have resulted in the fragmentation of wildlife habitats around the world, forcing animals to coexist with humans (Inskip & Zimmermann, 2009). Spiti Valley, a cold arid trans‐Himalayan landscape characterized by extreme environment and low species diversity (Shrotriya et al., 2022), is one such region threatened by development and tourism boom (Peaty, 2009). Tourism, coupled with unmanaged garbage and improper disposal of livestock carcasses, not only facilitates the red fox but also allows free‐roaming dogs, an introduced mesopredator, to thrive, possibly more than the native red fox (Ghoshal et al., 2016; Hughes & Macdonald, 2013). Despite large global populations of dogs and their negative interactions with native wildlife, only a handful of studies have information regarding the scale of conservation problems they may cause (Hughes & Macdonald, 2013). Further research into the effects of introduced mesopredator, that is, dogs on carnivore interspecific relationships can be refined by studying species' fine‐scale space use using GPS collars and including dietary analysis for dogs. We also emphasize the importance of longer‐term studies and data collection during the winter, a season of extreme resource scarcity that is expected to increase competition among these carnivores.

## AUTHOR CONTRIBUTIONS

**Priyanka Justa:** Conceptualization (supporting); formal analysis (equal); methodology (equal); writing – original draft (lead); writing – review and editing (equal). **Salvador Lyngdoh:** Conceptualization (lead); formal analysis (equal); funding acquisition (lead); investigation (lead); methodology (lead); project administration (lead); resources (lead); supervision (lead); writing – review and editing (lead).

## Supporting information


Appendix S1.

## Data Availability

The data that supports the findings of this study will be made available in the open repository upon acceptance. Data availability statement and the link to the Dryad repository having our datasets. DOI: https://doi.org/10.5061/dryad.573n5tbcj link to dryad dataset: https://datadryad.org/stash/share/D2_Asvua8J6PxTZytThYNJegQD6_lcojuZ2xBw4nwxk
