# Supplementary material for: Understanding carnivore interactions in a cold arid trans‐Himalayan landscape: What drives co‐existence patterns within predator guild along varying resource gradients?
Source: Ecol Evol. 2023 May 11;13(5):e10040. doi: 10.1002/ece3.10040 (PMC10173057; doi:10.1002/ece3.10040)
Supplement: Supplementary file 1 — Appendix S1. [file ECE3-13-e10040-s001.docx]

SUPPORTING INFORMATION


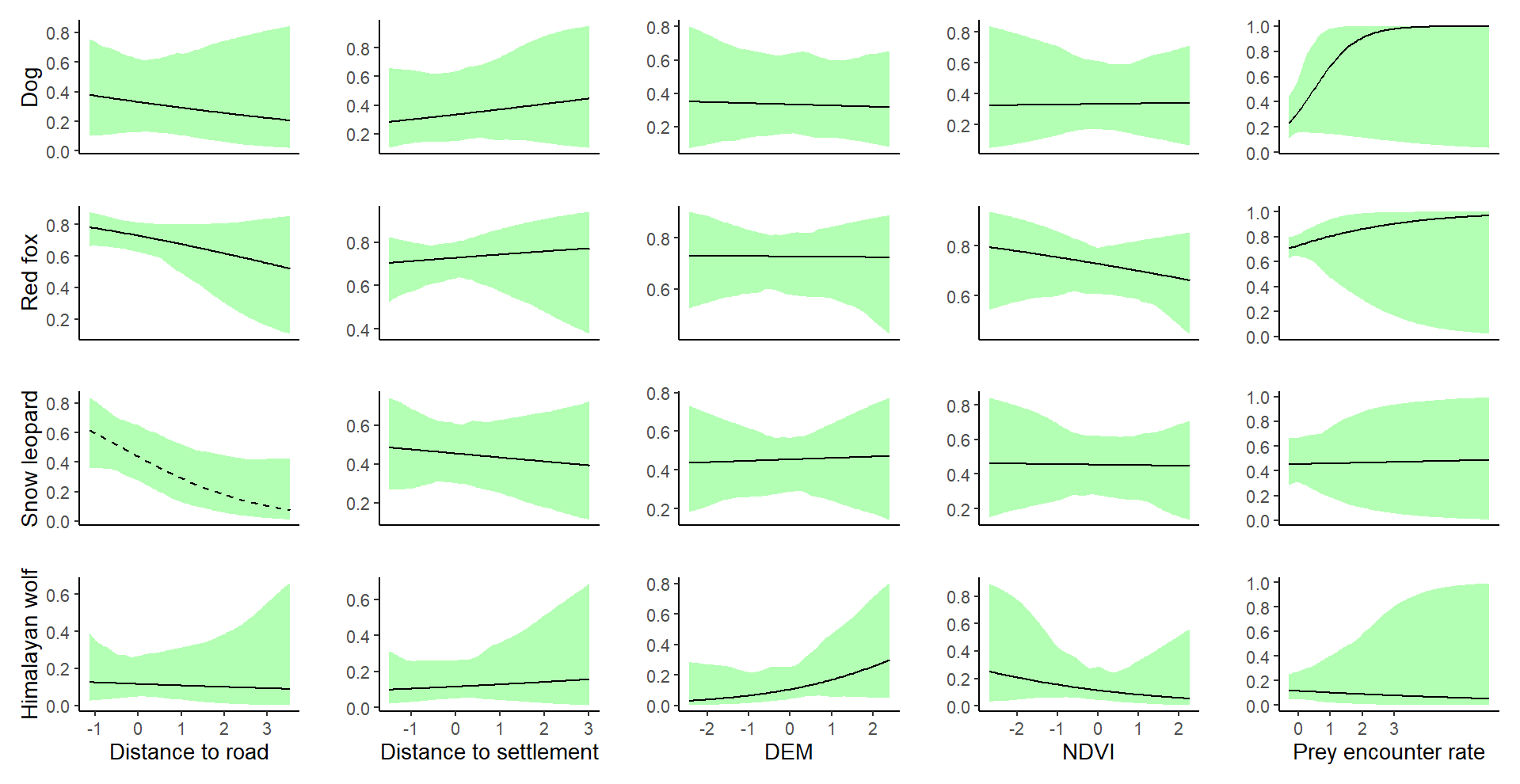


**Fig S1**. Marginal occupancy probabilities (on Y-axis) of predators in Lahaul and Spiti, H.P. as a function of covariates (on X-axis) representing the effect of one standard deviation change in covariate value. Dashed lines (indicating significant relationship) or Solid lines (indicating non-significant relationship) represent mean response, and green ribbons represent associated 95% credible intervals.

**Table S1**. Site covariates (all continuous variables) used for multispecies occupancy modelling in Spiti valley, Himachal Pradesh along with hypothesized effects of environmental covariates(+/-/N) on single-species occupancy for the study carnivore species and the source of covariates

| **Covariates** | **Dog** | **Red fox** | **Snow leopard** | **Wolf** | **Source** |
| --- | --- | --- | --- | --- | --- |
| Dist_settl | - | - | + | + | Euclidean distance calculated from settlement vector data of Open Street Maps |
|  |  |  |  |  |  |
| Dist_roadm | - | - | + | + | Euclidean distance calculated from vector road data of Open Street Maps |
|  |  |  |  |  |  |
| DEM (Digital Elevation Model) | - | N | + | + | ASTER (https://asterweb.jpl.nasa.gov/) |
|  |  |  |  |  |  |
| NDVI (Normalized Difference Vegetation Index) | + | + | - | - | Bhuvan (https://bhuvan.nrsc.gov.in/) |
|  |  |  |  |  |  |
| Abundance_Prey | + | + | + | + | Calculated from N-mixture models using count data |

+ positive, - negative, N neutral

**Table S2**: Log odds ratio for pairwise combinations of species (the log of the probability a species occupies a site divided by the probability it does not, conditional on the presence of an interacting species in Lahaul and Spiti, H.P. All second-order models are conditioned on all other species being absent.

| **Species pair** | **Estimate** | **SE** | **z** | **P(>\|z\|)** |
| --- | --- | --- | --- | --- |
| Red fox: Dog | 1.0 | 1.1 | 1.0 | 0.3 |
| Red fox: Snow leopard | 0.1 | 0.7 | 0.2 | 0.8 |
| Red fox: Wolf | -0.1 | 1.1 | -0.1 | 0.9 |
| Dog: Snow leopard | -0.3 | 1.0 | -0.3 | 0.8 |
| Dog: Wolf | 1.1 | 1.6 | 0.7 | 0.5 |
| Snow leopard: Wolf | -0.2 | 1.1 | -0.1 | 0.9 |

**Table S3.** Covariate coefficient estimates from the multi-species co-occurrence model for six species pairs (1 to 6) from camera-trap surveys in Lahaul and Spiti, H.P. The models have been arranged in ascending order of Akaike Information Criteria (AICc).

| 1. [Red fox: Dog] | Estimate | SE | z | P(>\|z\|) | nPars | AICc | Delta_AICc | AICcWt | Cum.Wt |
| --- | --- | --- | --- | --- | --- | --- | --- | --- | --- |
| *Dist_roadm* | **-1.2** | **0.8** | **-1.5** | **0.14** | **19** | **1066.4** | **0.0** | **0.3** | **0.3** |
|  |  |  |  |  |  |  |  |  |  |
| *Dist_roadm+*  *Dist_settl* | -1.6  0.4 | 0.8  0.5 | -2.0  0.9 | **0.04**  0.38 | 20 | 1068.7 | 2.3 | 0.1 | 0.4 |
|  |  |  |  |  |  |  |  |  |  |
| *Dist_roadm+*  *Small_prey* | -1.3  0.4 | 0.7  0.5 | -1.8  0.8 | **0.07**  0.41 | 20 | 1068.8 | 2.3 | 0.1 | 0.5 |
|  |  |  |  |  |  |  |  |  |  |
| *Small_prey* | 0.4 | 0.5 | 0.9 | 0.37 | 19 | 1069.0 | 2.5 | 0.1 | 0.6 |
|  |  |  |  |  |  |  |  |  |  |
| *Dist_roadm+*  *DEMmean* | -1.3  0.2 | 0.8  0.8 | -1.7  0.3 | **0.09**  0.76 | 20 | 1069.5 | 3.1 | 0.1 | 0.7 |
|  |  |  |  |  |  |  |  |  |  |
| *Dist_roadm+*  *NDVImean* | -1.2  -0.1 | 0.7  0.4 | -1.8  -0.2 | **0.07**  0.83 | 20 | 1069.6 | 3.1 | 0.1 | 0.8 |
|  |  |  |  |  |  |  |  |  |  |
| *DEMmean* | -0.3 | 0.5 | -0.5 | 0.62 | 19 | 1070.3 | 3.8 | 0.1 | 0.8 |
|  |  |  |  |  |  |  |  |  |  |
| *Dist_settl* | 0.1 | 0.4 | 0.3 | 0.75 | 19 | 1070.4 | 4.0 | 0.1 | 0.9 |
|  |  |  |  |  |  |  |  |  |  |
| *NDVImean* | -0.1 | 0.5 | -0.3 | 0.77 | 19 | 1070.4 | 4.0 | 0.0 | 0.9 |
|  |  |  |  |  |  |  |  |  |  |
| *DEMmean+*  *Small_prey* | -0.3  0.4 | 0.5  0.4 | -0.6  1.0 | 0.54  0.31 | 20 | 1071.7 | 5.3 | 0.0 | 0.9 |
|  |  |  |  |  |  |  |  |  |  |
| *Dist_settl+*  *Small_prey* | 0.2  0.5 | 0.4  0.5 | 0.5  0.9 | 0.63  0.34 | 20 | 1071.9 | 5.5 | 0.0 | 1.0 |
|  |  |  |  |  |  |  |  |  |  |
| *NDVImean+*  *Small_prey* | -0.2  0.4 | 0.4  0.4 | -0.4  1.0 | 0.67  0.33 | 20 | 1072.0 | 5.5 | 0.0 | 1.0 |
|  |  |  |  |  |  |  |  |  |  |
| *DEMmean+*  *NDVImean* | -0.3  -0.2 | 0.5  0.4 | -0.6  -0.5 | 0.53  0.59 | 20 | 1073.2 | 6.8 | 0.0 | 1.0 |
|  |  |  |  |  |  |  |  |  |  |
| *Dist_settl+*  *DEMmean* | 0.1  -0.3 | 0.5  0.6 | 0.3  -0.5 | 0.77  0.64 | 20 | 1073.4 | 6.9 | 0.0 | 1.0 |
|  |  |  |  |  |  |  |  |  |  |
| *Dist_settl+*  *NDVImean* | 0.1  -0.1 | 0.5  0.5 | 0.2  -0.2 | 0.81  0.84 | 20 | 1073.5 | 7.1 | 0.0 | 1.0 |

| 1. [Red fox: Snow leopard] | Estimate | SE | z | P(>\|z\|) | nPars | AICc | Delta_AICc | AICcWt | Cum.Wt |
| --- | --- | --- | --- | --- | --- | --- | --- | --- | --- |
| Small_prey | **0.9** | **0.6** | **1.4** | **0.15** | **19** | **1065.7** | **0.0** | **0.3** | **0.3** |
|  |  |  |  |  |  |  |  |  |  |
| DEMmean+  Small_prey | 0.5  0.8 | 0.3  0.6 | 1.4  1.5 | 0.16  0.13 | 20 | 1066.8 | 1.1 | 0.2 | 0.5 |
|  |  |  |  |  |  |  |  |  |  |
| DEMmean | 0.5 | 0.3 | 1.5 | 0.14 | 19 | 1068.2 | 2.6 | 0.1 | 0.6 |
|  |  |  |  |  |  |  |  |  |  |
| Dist_roadm+  Small_prey | 0.5  1.0 | 0.7  0.7 | 0.7  1.5 | 0.46  0.14 | 20 | 1068.3 | 2.6 | 0.1 | 0.7 |
|  |  |  |  |  |  |  |  |  |  |
| NDVImean+  Small_prey | 0.1  0.9 | 0.3  0.6 | 0.4  1.5 | 0.67  0.14 | 20 | 1068.7 | 3.0 | 0.1 | 0.8 |
|  |  |  |  |  |  |  |  |  |  |
| Dist_settl+  Small_prey | 0.1  0.9 | 0.3  0.6 | 0.2  1.4 | 0.83  0.17 | 20 | 1068.8 | 3.1 | 0.1 | 0.8 |
|  |  |  |  |  |  |  |  |  |  |
| NDVImean | 0.1 | 0.3 | 0.4 | 0.67 | 19 | 1070.3 | 4.7 | 0.0 | 0.9 |
|  |  |  |  |  |  |  |  |  |  |
| Dist_roadm | 0.3 | 0.7 | 0.4 | 0.69 | 19 | 1070.3 | 4.7 | 0.0 | 0.9 |
|  |  |  |  |  |  |  |  |  |  |
| Dist_settl | 0.1 | 0.3 | 0.4 | 0.70 | 19 | 1070.4 | 4.7 | 0.0 | 0.9 |
|  |  |  |  |  |  |  |  |  |  |
| DEMmean+  NDVImean | 0.5  0.1 | 0.3  0.3 | 1.5  0.3 | 0.14  0.77 | 20 | 1071.3 | 5.7 | 0.0 | 0.9 |
|  |  |  |  |  |  |  |  |  |  |
| Dist_roadm+  DEMmean | 0.0  0.5 | 0.7  0.3 | 0.1  1.4 | 0.96  0.15 | 20 | 1071.4 | 5.7 | 0.0 | 1.0 |
|  |  |  |  |  |  |  |  |  |  |
| Dist_settl+  DEMmean | 0.0  0.5 | 0.3  0.3 | 0.0  1.5 | 0.97  0.15 | 20 | 1071.4 | 5.7 | 0.0 | 1.0 |
|  |  |  |  |  |  |  |  |  |  |
| Dist_roadm+  NDVImean | 0.3  0.1 | 0.7  0.3 | 0.4  0.5 | 0.66  0.63 | 20 | 1073.3 | 7.6 | 0.0 | 1.0 |
|  |  |  |  |  |  |  |  |  |  |
| Dist_settl+  NDVImean | 0.1  0.1 | 0.3  0.3 | 0.3  0.4 | 0.75  0.71 | 20 | 1073.4 | 7.7 | 0.0 | 1.0 |
|  |  |  |  |  |  |  |  |  |  |
| Dist_roadm+  Dist_settl | 0.2  0.1 | 0.7  0.3 | 0.3  0.3 | 0.74  0.74 | 20 | 1073.4 | 7.8 | 0.0 | 1.0 |

| 1. [Red fox: Wolf] | Estimate | SE | z | P(>\|z\|) | nPars | AICc | Delta_AICc | AICcWt | Cum.Wt |
| --- | --- | --- | --- | --- | --- | --- | --- | --- | --- |
| Dist_roadm+  Small_prey | **-3.7**  **-9.4** | **2.0**  **8.2** | **-1.9**  **-1.1** | **0.06**  **0.25** | **20** | **1061.1** | **0.0** | **0.7** | **0.7** |
|  |  |  |  |  |  |  |  |  |  |
| Small_prey | -11.5 | 10.0 | -1.1 | 0.25 | 19 | 1065.3 | 4.2 | 0.1 | 0.8 |
|  |  |  |  |  |  |  |  |  |  |
| Dist_roadm | -2.7 | 1.8 | -1.5 | 0.14 | 19 | 1065.4 | 4.4 | 0.1 | 0.9 |
|  |  |  |  |  |  |  |  |  |  |
| Dist_roadm+  NDVImean | -3.5  -0.7 | 2.2  0.6 | -1.6  -1.3 | 0.10  0.21 | 20 | 1066.8 | 5.7 | 0.0 | 0.9 |
|  |  |  |  |  |  |  |  |  |  |
| Dist_settl+  Small_prey | 0.3  -11.4 | 0.5  9.8 | 0.5  -1.2 | 0.60  0.25 | 20 | 1068.2 | 7.1 | 0.0 | 0.9 |
|  |  |  |  |  |  |  |  |  |  |
| DEMmean+  Small_prey | 0.2  -11.6 | 0.9  10.1 | 0.2  -1.2 | 0.86  0.25 | 20 | 1068.4 | 7.3 | 0.0 | 0.9 |
|  |  |  |  |  |  |  |  |  |  |
| Dist_roadm+  DEMmean | -3.0  0.4 | 2.0  1.0 | -1.5  0.4 | 0.14  0.67 | 20 | 1068.4 | 7.3 | 0.0 | 0.9 |
|  |  |  |  |  |  |  |  |  |  |
| NDVImean+  Small_prey | 0.2  -11.6 | 0.9  10.1 | 0.2  -1.2 | 0.86  0.25 | 20 | 1068.4 | 7.3 | 0.0 | 1.0 |
|  |  |  |  |  |  |  |  |  |  |
| Dist_roadm+  Dist_settl | -2.7  0.1 | 1.8  0.5 | -1.5  0.2 | 0.14  0.86 | 20 | 1068.6 | 7.5 | 0.0 | 1.0 |
|  |  |  |  |  |  |  |  |  |  |
| NDVImean | -0.5 | 0.6 | -0.8 | 0.45 | 19 | 1069.8 | 8.7 | 0.0 | 1.0 |
|  |  |  |  |  |  |  |  |  |  |
| DEMmean | -0.1 | 0.9 | -0.1 | 0.89 | 19 | 1070.5 | 9.4 | 0.0 | 1.0 |
|  |  |  |  |  |  |  |  |  |  |
| Dist_settl | 0.0 | 0.5 | 0.0 | 0.97 | 19 | 1070.5 | 9.4 | 0.0 | 1.0 |
|  |  |  |  |  |  |  |  |  |  |
| Dist_settl+  NDVImean | 0.0  -0.5 | 0.5  0.7 | 0.1  -0.7 | 0.93  0.47 | 20 | 1073.0 | 11.9 | 0.0 | 1.0 |
|  |  |  |  |  |  |  |  |  |  |
| DEMmean+  NDVImean | 0.0  -0.5 | 1.0  0.6 | 0.0  -0.7 | 0.99  0.46 | 20 | 1073.0 | 11.9 | 0.0 | 1.0 |
|  |  |  |  |  |  |  |  |  |  |
| Dist_settl+  DEMmean | 0.0  -0.1 | 0.5  0.9 | 0.0  -0.1 | 0.98  0.90 | 20 | 1073.7 | 12.6 | 0.0 | 1.0 |

| 1. [Dog: Snow leopard] | Estimate | SE | z | P(>\|z\|) | nPars | AICc | Delta_AICc | AICcWt | Cum.Wt |
| --- | --- | --- | --- | --- | --- | --- | --- | --- | --- |
| DEMmean | -304.1 | 435.6 | -0.7 | 0.49 | 19 | 1055.6 | 0.0 | 0.3 | 0.3 |
|  |  |  |  |  |  |  |  |  |  |
| Dist_roadm+  NDVImean | **-10.9**  **-1.5** | **3.8**  **0.7** | **-2.8**  **-2.3** | **0.00**  **0.02** | **20** | **1056.0** | **0.5** | **0.2** | **0.5** |
|  |  |  |  |  |  |  |  |  |  |
| DEMmean+  NDVImean | -75.8  -1.5 | 61.4  0.8 | -1.2  -1.9 | 0.22  **0.05** | 20 | 1056.1 | 0.5 | 0.2 | 0.7 |
|  |  |  |  |  |  |  |  |  |  |
| Dist_settl+  DEMmean | 0.8  -96.4 | 0.5  NaN | 1.7  NaN | 0.10  NaN | 20 | 1056.9 | 1.3 | 0.1 | 0.9 |
|  |  |  |  |  |  |  |  |  |  |
| DEMmean+  Large_prey | -304.3  -0.4 | 434.8  1.9 | -0.7  -0.2 | 0.48  0.84 | 20 | 1058.7 | 3.1 | 0.1 | 0.9 |
|  |  |  |  |  |  |  |  |  |  |
| Dist_roadm | -6.3 | 2.6 | -2.4 | **0.01** | 19 | 1058.9 | 3.4 | 0.1 | 1.0 |
|  |  |  |  |  |  |  |  |  |  |
| Dist_roadm+  DEMmean | -7.7  0.9 | 3.1  0.8 | -2.5  1.1 | **0.01**  0.27 | 20 | 1060.9 | 5.4 | 0.0 | 1.0 |
|  |  |  |  |  |  |  |  |  |  |
| Dist_roadm+  Large_prey | -7.5  1.0 | 3.3  1.0 | -2.2  1.0 | **0.03**  0.31 | 20 | 1061.3 | 5.7 | 0.0 | 1.0 |
|  |  |  |  |  |  |  |  |  |  |
| Dist_roadm+  Dist_settl | -6.8  0.3 | 2.9  0.6 | -2.3  0.5 | **0.02**  0.61 | 20 | 1061.9 | 6.3 | 0.0 | 1.0 |
|  |  |  |  |  |  |  |  |  |  |
| NDVImean | -0.8 | 0.8 | -1.0 | 0.32 | 19 | 1069.7 | 14.1 | 0.0 | 1.0 |
|  |  |  |  |  |  |  |  |  |  |
| Large_prey | -0.2 | 0.5 | -0.3 | 0.74 | 19 | 1070.4 | 14.8 | 0.0 | 1.0 |
|  |  |  |  |  |  |  |  |  |  |
| Dist_settl | -0.2 | 0.9 | -0.3 | 0.79 | 19 | 1070.4 | 14.8 | 0.0 | 1.0 |
|  |  |  |  |  |  |  |  |  |  |
| Dist_settl+  NDVImean | -0.8  -1.3 | 0.8  1.1 | -1.1  -1.2 | 0.28  0.21 | 20 | 1071.4 | 15.8 | 0.0 | 1.0 |
|  |  |  |  |  |  |  |  |  |  |
| NDVImean+  Large_prey | -0.7  -0.1 | 0.8  0.6 | -0.9  -0.2 | 0.34  0.82 | 20 | 1072.8 | 17.2 | 0.0 | 1.0 |
|  |  |  |  |  |  |  |  |  |  |
| Dist_settl+  Large_prey | -0.2  -0.2 | 0.9  0.5 | -0.3  -0.3 | 0.78  0.74 | 20 | 1073.5 | 17.9 | 0.0 | 1.0 |

| 1. [Dog: Wolf] | Estimate | SE | z | P(>\|z\|) | nPars | AICc | Delta_AICc | AICcWt | Cum.Wt |
| --- | --- | --- | --- | --- | --- | --- | --- | --- | --- |
| Dist_roadm+  NDVImean | **-10.2**  **-2.2** | **4.5**  **1.0** | **-2.3**  **-2.1** | **0.02**  **0.03** | **20** | **1066.5** | **0.0** | **0.2** | **0.2** |
|  |  |  |  |  |  |  |  |  |  |
| Dist_settl+  DEMmean | 1.5  -3.2 | 0.9  1.4 | 1.7  -2.3 | 0.09  0.02 | 20 | 1066.9 | 0.4 | 0.2 | 0.4 |
|  |  |  |  |  |  |  |  |  |  |
| DEMmean | -2.3 | 1.2 | -2.0 | 0.05 | 19 | 1067.2 | 0.7 | 0.2 | 0.5 |
|  |  |  |  |  |  |  |  |  |  |
| Dist_roadm | -2.2 | 2.2 | -1.0 | 0.31 | 19 | 1068.1 | 1.6 | 0.1 | 0.6 |
|  |  |  |  |  |  |  |  |  |  |
| Large_prey | -1.3 | 1.5 | -0.8 | 0.40 | 19 | 1069.1 | 2.6 | 0.1 | 0.7 |
|  |  |  |  |  |  |  |  |  |  |
| DEMmean+  NDVImean | -2.1  -1.2 | 1.1  1.2 | -1.8  -0.9 | **0.07**  0.35 | 20 | 1069.2 | 2.7 | 0.1 | 0.8 |
|  |  |  |  |  |  |  |  |  |  |
| NDVImean | -0.6 | 0.9 | -0.7 | 0.47 | 19 | 1069.7 | 3.2 | 0.0 | 0.8 |
|  |  |  |  |  |  |  |  |  |  |
| Dist_roadm+  DEMmean | -1.4  -2.1 | 2.7  1.2 | -0.5  -1.8 | 0.59  **0.08** | 20 | 1069.9 | 3.4 | 0.0 | 0.8 |
|  |  |  |  |  |  |  |  |  |  |
| Dist_roadm+  Large_prey | -2.6  -1.6 | 2.4  1.6 | -1.1  -1.0 | 0.27  0.31 | 20 | 1070.0 | 3.5 | 0.0 | 0.9 |
|  |  |  |  |  |  |  |  |  |  |
| DEMmean+  Large_prey | -2.1  -1.2 | 1.2  3.0 | -1.7  -0.4 | **0.08**  0.69 | 20 | 1070.0 | 3.5 | 0.0 | 0.9 |
|  |  |  |  |  |  |  |  |  |  |
| Dist_settl | 0.3 | 0.8 | 0.4 | 0.67 | 19 | 1070.3 | 3.8 | 0.0 | 0.9 |
|  |  |  |  |  |  |  |  |  |  |
| Dist_roadm+  Dist_settl | -2.5  0.3 | 2.7  0.8 | -0.9  0.3 | 0.35  0.74 | 20 | 1071.1 | 4.6 | 0.0 | 1.0 |
|  |  |  |  |  |  |  |  |  |  |
| NDVImean+  Large_prey | -0.3  -1.2 | 0.5  1.4 | -0.7  -0.8 | 0.50  0.41 | 20 | 1071.7 | 5.2 | 0.0 | 1.0 |
|  |  |  |  |  |  |  |  |  |  |
| Dist_settl+  Large_prey | 0.3  -1.3 | 1.0  1.7 | 0.3  -0.8 | 0.78  0.44 | 20 | 1072.2 | 5.7 | 0.0 | 1.0 |
|  |  |  |  |  |  |  |  |  |  |
| Dist_settl+  NDVImean | 0.4  -0.7 | 0.7  0.8 | 0.5  -0.8 | 0.63  0.43 | 20 | 1072.6 | 6.1 | 0.0 | 1.0 |

| 1. [Snow leopard: Wolf] | Estimate | SE | z | P(>\|z\|) | nPars | AICc | Delta_AICc | AICcWt | Cum.Wt |
| --- | --- | --- | --- | --- | --- | --- | --- | --- | --- |
| Dist_roadm+  DEMmean | -591.7  228.0 | 4293.7  864.8 | -0.1  0.3 | 0.89  0.79 | 20 | 1058.5 | 0.0 | 0.5 | 0.5 |
|  |  |  |  |  |  |  |  |  |  |
| Dist_roadm+  NDVImean | **-11.4**  **-1.5** | **3.8**  **0.6** | **-3.0**  **-2.4** | **0.00**  **0.02** | **20** | **1059.3** | **0.8** | **0.4** | **0.9** |
|  |  |  |  |  |  |  |  |  |  |
| Dist_roadm | -6.4 | 2.5 | -2.6 | 0.01 | 19 | 1062.4 | 4.0 | 0.1 | 1.0 |
|  |  |  |  |  |  |  |  |  |  |
| Dist_roadm+  Large_prey | -7.4  1.0 | 3.2  1.0 | -2.3  1.0 | 0.02  0.31 | 20 | 1064.8 | 6.3 | 0.0 | 1.0 |
|  |  |  |  |  |  |  |  |  |  |
| Dist_roadm+  Dist_settl | -7.2  0.5 | 2.9  0.6 | -2.5  0.7 | 0.01  0.47 | 20 | 1065.1 | 6.6 | 0.0 | 1.0 |
|  |  |  |  |  |  |  |  |  |  |
| DEMmean | 1.6 | 1.2 | 1.4 | 0.17 | 19 | 1068.4 | 9.9 | 0.0 | 1.0 |
|  |  |  |  |  |  |  |  |  |  |
| Dist_settl+  DEMmean | -0.9  2.1 | 0.8  1.4 | -1.1  1.5 | 0.28  0.12 | 20 | 1070.2 | 11.7 | 0.0 | 1.0 |
|  |  |  |  |  |  |  |  |  |  |
| Dist_settl | -0.3 | 0.8 | -0.4 | 0.67 | 19 | 1070.3 | 11.9 | 0.0 | 1.0 |
|  |  |  |  |  |  |  |  |  |  |
| Large_prey | -0.3 | 1.0 | -0.3 | 0.74 | 19 | 1070.3 | 11.9 | 0.0 | 1.0 |
|  |  |  |  |  |  |  |  |  |  |
| NDVImean | 0.2 | 0.7 | 0.3 | 0.79 | 19 | 1070.4 | 12.0 | 0.0 | 1.0 |
|  |  |  |  |  |  |  |  |  |  |
| DEMmean+  NDVImean | 1.9  0.5 | 1.2  0.7 | 1.5  0.7 | 0.13  0.48 | 20 | 1071.0 | 12.5 | 0.0 | 1.0 |
|  |  |  |  |  |  |  |  |  |  |
| DEMmean+  Large_prey | 1.7  -0.3 | 1.2  1.1 | 1.4  -0.3 | 0.17  0.79 | 20 | 1071.4 | 13.0 | 0.0 | 1.0 |
|  |  |  |  |  |  |  |  |  |  |
| Dist_settl+  Large_prey | -0.3  -0.2 | 0.8  0.9 | -0.4  -0.3 | 0.72  0.78 | 20 | 1073.4 | 14.9 | 0.0 | 1.0 |
|  |  |  |  |  |  |  |  |  |  |
| Dist_settl+  NDVImean | -0.4  0.2 | 0.8  0.8 | -0.4  0.3 | 0.65  0.75 | 20 | 1073.4 | 14.9 | 0.0 | 1.0 |
|  |  |  |  |  |  |  |  |  |  |
| NDVImean+  Large_prey | 0.2  -0.3 | 0.7  1.0 | 0.3  -0.3 | 0.78  0.74 | 20 | 1073.4 | 15.0 | 0.0 | 1.0 |

*Dist_roadm: average distance to road in a grid; Dist_settl: average distance to settlement in a grid; DEMmean: DEM (Digital Elevation Model) NDVImean: NDVI (Normalized Difference Vegetation Index); Small_prey: Photo capture rates of pika and woolly hare; Large_prey: Photo capture rates of blue sheep, ibex and livestock
